# A novel algorithm developed using machine learning and a J-ACCESS database can estimate defect scores from myocardial perfusion single-photon emission tomography images

**DOI:** 10.1007/s12149-024-01971-z

**Published:** 2024-08-29

**Authors:** Keisuke Kiso, Kenichi Nakajima, Yukitaka Nimura, Tsunehiko Nishimura

**Affiliations:** 1 Department of Diagnostic Radiology, Tohoku University Hospital, 1-1 Seiryo-Machi, Aoba-Ku, Sendai, 980-8574 Japan; 2 Department of Radiology, Sendai Medical Imaging Center, Sendai, Japan; 3 Department of Nuclear Medicine/Functional Imaging and Artificial Intelligence, Kanazawa University, 13-1 Takara-machi, Kanazawa, 920‑8640 Japan; 4 Locus Logic, Inc., Nagoya, Japan; 5 Graduate School of Medical Science, Kyoto Prefectural University of Medicine, Kyoto, Japan

**Keywords:** Coronary artery disease, Myocardial perfusion imaging, Artificial intelligence, Quantification

## Abstract

**Background:**

Stress myocardial perfusion single-photon emission computed tomography (SPECT) imaging (MPI) has been used to diagnose and predict the prognoses of patients with coronary artery disease (CAD). An ongoing multicenter collaboration established a Japanese database (J-ACCESS) in 2001 that includes a risk model and expert interpretations. The present study aimed to develop a novel algorithm using machine learning (ML) and resources from the J-ACCESS database to aid SPECT image interpretation.

**Methods:**

We analyzed data from 1288 patients in J-ACCESS 3 and 4 databases. Three-dimensional (3D) stereoscopic images of left ventricular myocardial perfusion were reconstructed with linear transformation from the original short-axis data. Segments were extracted from U-Net, then features were extracted from each segment during the ML process. We estimated segmental scores based on weighted features obtained from fully connected layers. Correlations between segment scores interpreted by nuclear cardiology experts and estimated by ML were evaluated using a 17-segment model, summed stress (SSS), summed rest (SRS), and summed difference (SDS) scores, and ratios (%) of summed different scores (%SDS).

**Results:**

The complete concordance rate of scores assessed by the experts and estimated by ML was 79.6%. The underestimated and overestimated rates were 10.3% and 10.0%, respectively. Associations between defect scores assessed by experts and ML were close, with correlation coefficients (*r*) of 0.923, 0.917, 0.842 and 0.853 for SSS, SRS, SDS, %SDS, respectively (*p* < 0.0001 for all).

**Conclusions:**

We created a new algorithm to estimate MPI scores using ML and the J-ACCESS database. This algorithm should provide accurate MPI interpretation even in facilities without specialist nuclear cardiologists, and might facilitate therapeutic decision-making and predict prognoses.

## Introduction

Stress myocardial perfusion single-photon emission computed tomography (SPECT) imaging (MPI) has been used to measure the extent and severity of inducible ischemia in patients with coronary artery disease (CAD). Prediction of prognosis in patients with CAD is important for selecting subsequent therapy such as reperfusion or pharmaceutical administration [1–3]. The Japanese Assessment of Cardiac Events and Survival Study by Quantitative Gated SPECT (J-ACCESS investigation) database was established in 2001 [4]. That study investigated the ability of myocardial perfusion and cardiac function determined by ECG gated-MPI to predict event rates [5]. A risk model was then created for patients with suspected ischemic heart disease [6] using the J-ACCESS database. The relationship between abnormal myocardial perfusion and prognosis among patients with CAD comorbidities, such as diabetes mellitus (DM) and chronic kidney disease (CKD), was clarified in J-ACCESS 2 [7–9] and 3 [10–12]. The importance of assessing reductions in the ischemic burden was evaluated by stress MPI to predict prognoses after reperfusion or optimal medical therapy in J-ACCESS 4 [13, 14]. The clinical implications of the J-ACCESS findings among patients with heart failure have also been summarized [15].

Despite the value of MPS, MPI has gradually decreased in Japanese clinical practice, whereas the prevalence of coronary CT angiography and cardiac MRI has increased (Japanese Registry Of All cardiac and vascular Diseases (JROAD): Annual Report 2022). Reasons for underutilized MPI might be blurred images and dependence on operator experience unlike CT and MRI.

Several groups have attempted to improve the accuracy of diagnostic imaging using artificial intelligence (AI) [16–22]. The J-ACCESS 3 and 4 database includes segmental scores evaluated by nuclear cardiology experts who belong to the Image Interpretation Committee (IIC) of J-ACCESS investigations. Therefore, we aimed to develop a novel ML algorithm using the SPECT image and interpretation resources of J-ACCESS 3 and 4 that would enable non-specialists to evaluate MPI scores as well as nuclear cardiology experts.

## Methods

### Patients

Among 1343 patients enrolled in the J-ACCESS 3 and 4 databases (*n* = 549 and 794, respectively), 55 patients, whose data could not be loaded to ML process due to damaged original DICOM (Digital Imaging and Communications in Medicine) format, were excluded. We subsequently analyzed data derived from 1288 (male, 906; female, 382) patients. Stress and rest MPI data were analyzed separately; thus, 2596 patients were included for ML. The inclusion and exclusion criteria in J-ACCESS 3 and 4 are described elsewhere [10, 14].

### Myocardial perfusion SPECT

The protocols for stress/rest MPI with ^99m^Tc-tetrofosmin used in J-ACCESS 3 and 4, are described elsewhere [10, 14]. Pharmacological stress was induced by adenosine using standard MPI protocols at each hospital. However, the number and type of detectors (Anger or semiconductor), image acquisition conditions such as matrix size, number of steps and camera rotation ranges, were not regulated. The acquisition conditions, however, followed the standard protocol at each institution. Only non-gated perfusion data were utilized in this study.

### Expert evaluation of MPI scoring registered in J-ACCESS 3 and 4 databases

Short-axis, vertical and horizontal long-axis views were generated by MPI using a standard verified processing protocol [5, 12, 14, 23]. All reconstructed short-axis data created at each site were sent to the J-ACCESS core laboratory (Osaka, Japan) in the DICOM format.

Nuclear cardiology experts evaluated digital images using a 17-segment model to increase defect scoring objectivity [10, 13]. Perfusion in segments was respectively scored as 0, 1, 2, 3, and 4 representing normal, mildly, moderately, severely reduced, and absent. Summed stress (SSS), rest (SRS) and difference (SDS) scores for all 17 segments were calculated.

### Datasets

Data from the 2,576 patients were assigned to the following datasets for ML.

The training dataset comprised imaging data from 43,112 segments derived from 2536 patients (male, *n* = 1792, 30,464 segments; female, *n* = 744, 12,648 segments). The validation dataset consisted of images that were derived from 10 male and 10 female patients and subdivided into 170 segments each. In this dataset, 11 patients had significant myocardial ischemia (SSS ≥ 4), and the culprit coronary artery was left anterior descending (LAD) in five patients, left circumflex (LCX) in four patients, and right coronary artery (RCA) in two patients. This dataset was used to mitigate the risk of overfitting to the training data. We stopped updating parameters if the estimation accuracy did not improve beyond 200 epochs. The test dataset also comprised images derived from 10 males and 10 females subdivided into 170 segments each to evaluate the ML.

The interchangeable roles of the validation and test datasets increased the number of trials required to enhance the reliability of the experimental results. Overall, the test phase of the ML model included 40 patients (680 segments).

### Image processing and ML methods

#### Image processing

We reconstructed three-dimensional (3D) stereoscopic images of left ventricular myocardial perfusion with linear transformation from original short-axis data of the J-ACCESS 3 and 4 databases.

Image dimensions were standardized to 64 × 64 × 64 pixels, with 3 × 3 × 3 mm^3^ intervals. During the training phase, we incorporated a data augmentation technique that randomly shifted images left, right, up and down by a maximum of two pixels to minimize positional bias in the dataset.

**Fig. 1:**
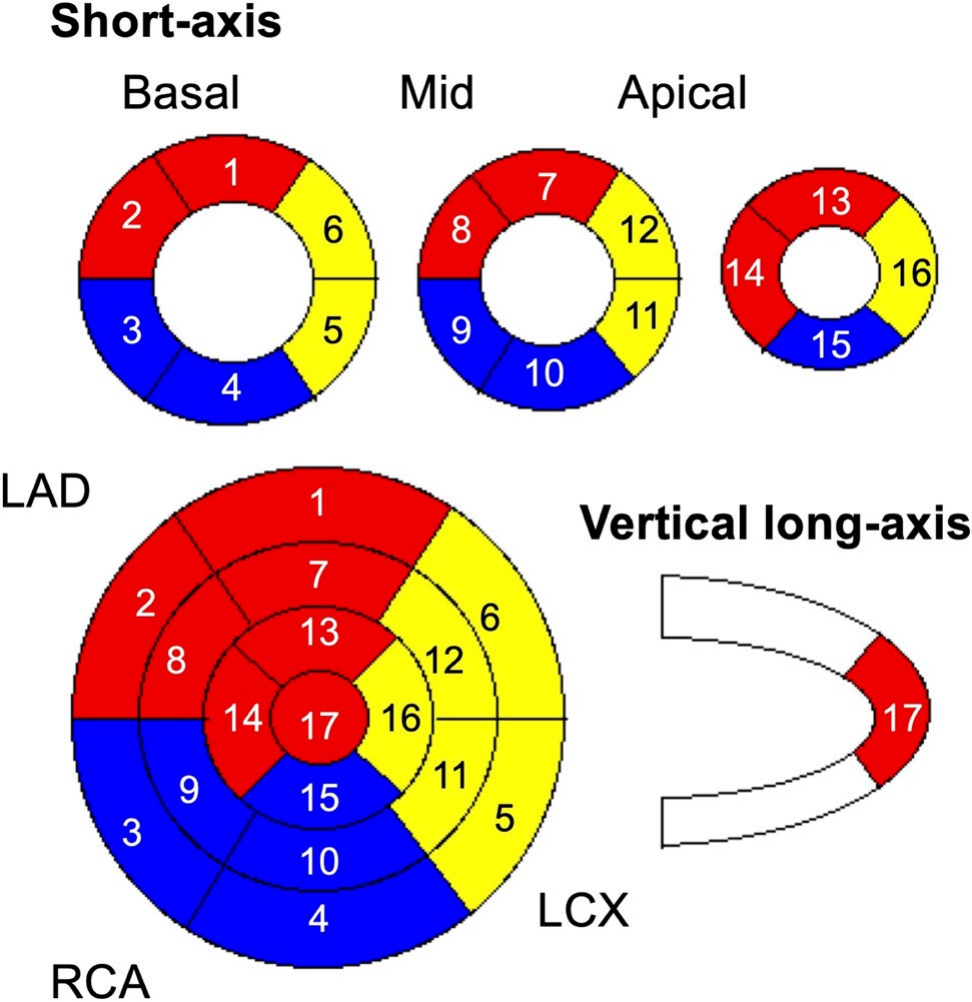
Numbers in 17-segment model and corresponding coronary artery territories. Red, yellow and blue segments represent territories of left anterior descending artery (LAD), left circumflex artery (LCX), and right coronary artery (RCA), respectively

We used the 17-segment model recommended by the American Heart Association [24] for segmental analysis. Figure 1 shows numbered segments and the territories of the coronary arteries. The segments are numbered 1, 2, 7, 8, 13, 14, and 17; 5, 6, 11, 12, and 16; and 3, 4, 9, 10, and 15 and correspond to regions of the LAD, LCX and RCA arteries, respectively.

#### Machine learning with neural network analysis

**Fig. 2:**
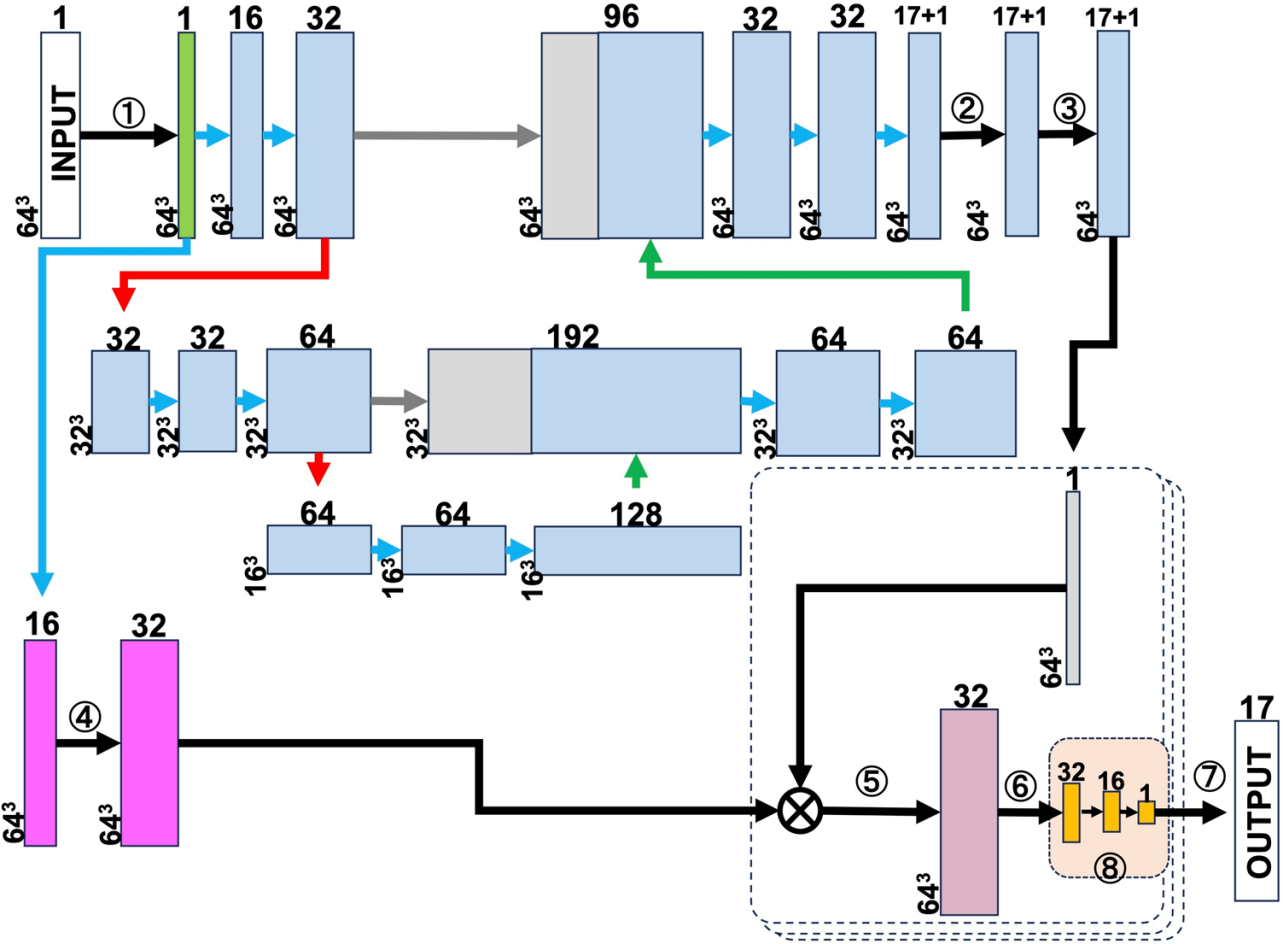
Configuration of neural network. Rectangle in upper left corner labeled as “input image” represents initial data used for machine learning and that in right lower corner labeled “output of segmental scores” represents final analytical outcome. Others are feature maps generated by convolution process of our neural network. Superscript and subscript values in rectangles represent number of channels and size of feature map. Blue and gray rectangles in upper and middle rows are parts of “U-Net”, which extracts myocardial segments from input data. Pink rectangles in lower left portion are part of convolutional neural network (CNN) architecture and were designed for feature value extraction. Orange rectangles in lower right shows segmental score estimated by fully connected layers. This calculation is based on results of multiplying feature values determined by CNN and probabilities of segment position extracted by U-Net. Part surrounded by dotted line shows processes in each segment. Blue, gray, red and green arrows respectively show convolution 3 × 3, batch normalization ReLU, copy, max pool 2 × 2, and transposed convolution 2 × 2. Encircled numbers represent image normalization (1), Softmax (2), convolution 3 × 3 (3), convolution 7 × 7 (4), multiplication (5), spacewise max pool, (6) append channels (7), fully connected layers (shared weight) (8)

Figure 2 shows the configuration of the neural network. Teaching data consisted of combinations of images and segmental scores evaluated by expert nuclear cariologists. Imaging data and estimated segmental defect scores were respectively input into and output from the neural network.

The parameters of the neural network were updated during ML to minimize the Log-Cosh loss function between the estimated and actual segmental scores output by the neural network and those determined by the experts.

Our neural network model was designed to directly estimate segmental scores based on reconstructed 3D images (Fig. 2). The process began with segment extraction, which involved estimating the position and region of each segment from 3D images reconstructed using U-Net (26). Features were extracted from images using a concurrent convolutional neural network (CNN). These features were then weighted according to the segments identified by U-Net, thus masking the entire feature set that corresponded to the target segment. Finally, segmental scores were estimated from the weighted features using fully connected layers (FCL).

The training, validation and test datasets comprised 3D images with segmental scores assessed by the experts. The model parameters were refined during the training phase to minimize the Log-Cosh loss function applied to the training dataset. This loss function calculates the hyperbolic cosine of error between the estimated and correct scores.

We evaluated the mean squared error (MSE) of the validation dataset to mitigate overfitting to the training dataset. The MSE metric was used to stop updating model parameters if estimating accuracy did not improve beyond 200 epochs. Determining the optimal number of training epochs that are critical model hyperparameters, presented a significant challenge. It was established with careful consideration before training started.

Correlations between the correct and estimated segmental scores were assessed across 17 segments, specific regions (such as the basal region), and summed scores (SSS, SRS, SDS, and %SDS) using the test datasets. This evaluation was to verify the ability of the models to accurately predict scores at the individual segment level and for broader areas.

### Statistical analysis

Associations were evaluated using Pearson correlation analyses. All test values with *p* ≤ 0.05 were considered statistically significant. All data were analyzed using the statistical analysis system (SAS) Version 9.3 (SAS Institute Inc., Cary, NC, USA).

## Results

### Breakdown of segmental scores derived from J-ACCESS databases

Table 1 shows the breakdown of segmental perfusion scores in each dataset assessed by experts. As the score increased, numbers decreased. Segments without and minor defect (score 0–1) comprised 96% and those with moderate and severe defect (score 2–4) comprised 4% of the total segments.

**Table 1:** Breakdown of segmental scores in datasets derived from J-ACCESS databases

Dataset		Scores					All
		0	1	2	3	4	
Training	M	27,329	1838	1001	206	90	30,464
	F	11,921	481	189	47	10	12,648
Validation A (test B)	M	135	19	7	5	4	170
	F	140	12	13	1	4	170
Test A (validation B)	M	132	22	8	6	2	170
	F	142	13	8	3	4	170
All		39,799	2385	1226	268	114	43,792

*F* female, *M* male

### Correlations of scores between experts and ML

Table 2 shows the comparison of scores assessed by experts and estimated by the neural network in 40 tests of A and B (validation A). The complete concordance rate was 79.6%, and the underestimated and overestimated rates were 10.3% and 10.0%, respectively.

**Table 2:** Correlations between scores estimated by nuclear cardiology experts and neural network

Expert scores	Estimated scores				
	0	1	2	3	4
0	495	45	9	0	0
1	29	27	8	2	0
2	4	16	10	5	1
3	0	2	6	6	1
4	0	0	1	10	3

### Concordance rates in 17-segment models

Table 3 shows concordance rates between the neural network and experts in 17-segment model. The segment numbers and corresponding coronary artery territories are shown in Fig. 1. The rates were < 70% in segments 4, 5, 10, 11 in the mid and basal inferolateral segments.

**Table 3:** Concordance rates in one-by-one comparisons of 17 segments (total segments per segment: *n* = 40)

Regions	Segments 1‒17					
Basal						
Segment numbers	1	2	3	4	5	6
Concordant segments (*n*)	38	34	35	26	26	35
Concordance rate (%)	95.0	85	87.5	65.0	65.0	87.5
Mid						
Segment numbers	7	8	9	10	11	12
Concordant segments (*n*)	35	33	36	25	27	34
Concordance rate (%)	87.5	82.5	90.0	62.5	67.5	85.0
Apical						
Segment numbers	14	15	16	17		
Concordant segments (*n*)	33	33	31	28		
Concordance rate (%)	82.5	82.5	77.5	70.0		

### Concordance rates in basal regions and coronary artery territories

Table 4 shows that the concordance rate was 79.6% for segments 1‒17 (80.8% and 86.3% for basal and basal septal segments 1‒6 and 2‒3, respectively). The rates in the coronary territories were 83.2% (LAD), 76.5% (LCX), and 77.5% (RCA), respectively (Table 5).

**Table 4:** Concordance of defect scores in overall and basal segments

	Overall	Basal regions	Basal septum
Concordant segments (*n*)	541	194	69
Concordance rate (%)	79.6	80.8	86.3

Total segments in basal regions 1‒6 in Fig. 1, *n* = 240; basal septum 2 and 3, *n* = 80. Overall total segments, *n* = 680

**Table 5:** Concordance rates in coronary artery territories

	RCA	LAD	LCX
Concordant segments (*n*)	155	233	153
Concordance rate (%)	77.5	83.2	76.5

Total segments in left anterior descending artery (LAD), 280; left circumflex artery (LCX), 200; right coronary artery (RCA), 200

### Estimation accuracy for maximum perfusion defect score in each test

Table 6 shows the estimation accuracy calculated as the MSE for maximum perfusion defect scores in tests A and B. Increasing scores indicated decreased accuracy. Moreover, accuracy was worse in the female, than in the male dataset.

**Table 6:** Estimation accuracy (MSE) of maximum perfusion defect scores in each test

Dataset	Max score					Mean
	0	1	2	3	4	
All	0.051	0.100	0.129	0.266	0.708	0.251
All (M)	0.093	0.130	0.144	0.319	0.409	0.275
All (F)	0.007	0.071	0.113	0.212	1.006	0.219
Male	0.068	0.066	0.136	0.304	0.327	0.180
Female	0.118	0.116	0.291	0.406	1.842	0.554

*MSE* mean squared error

### Correlation in SSS/SRS/SDS/%SDS between expert interpretation and neural network

**Fig. 3:**
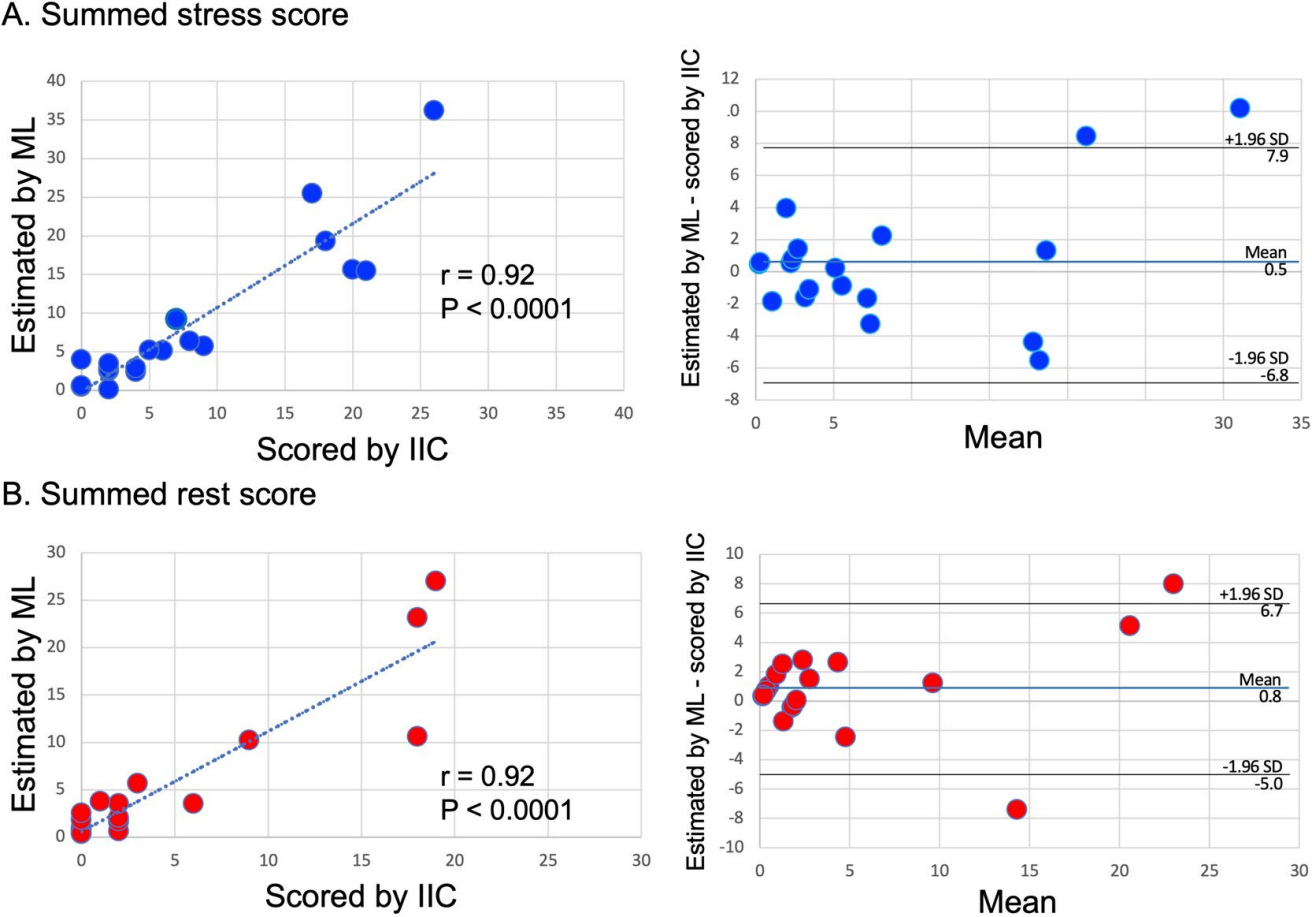
Correlations of summed stress and rest scores (SSS, SRS) between expert imaging (image interpretation committee, IIC) and machine learning (ML) interpretations

**Fig. 4:**
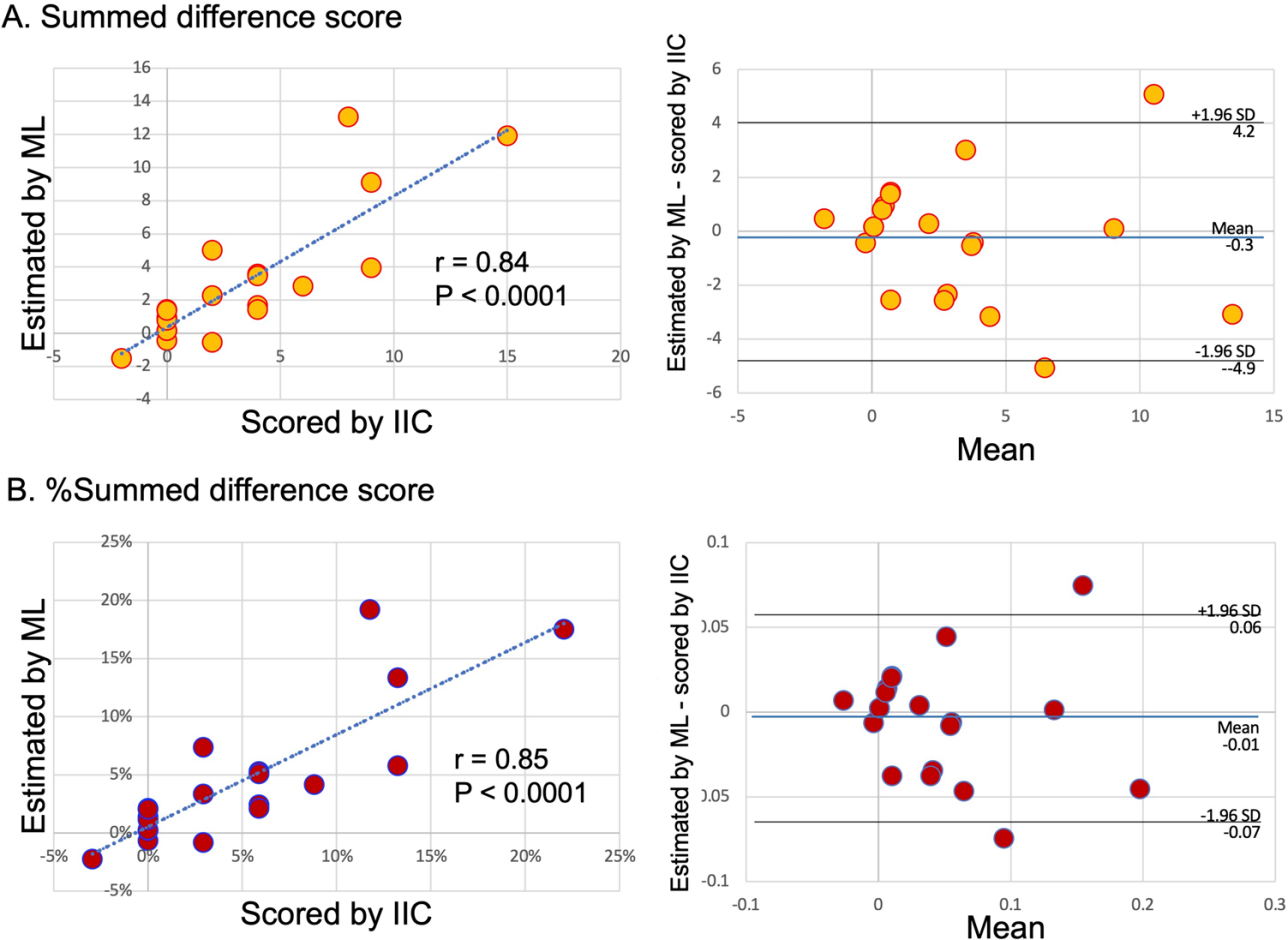
Correlations of summed difference scores (SDS) and %SDS between expert image (image interpretation committee, IIC) and machine learning (ML) interpretations

The results of the Bland–Altman plots of correlations (*r*) between SSS, SRS, SDS and %SDS interpreted by experts and by the neural network were *r* = 0.923, *r* = 0.917, *r* = 0.842, *r* = 0.853, respectively (*p* < 0.0001 for all; Figs. 3 and 4).

### The prognostic significance of MPI values evaluated by the ML-based algorithm

**Fig. 5:**
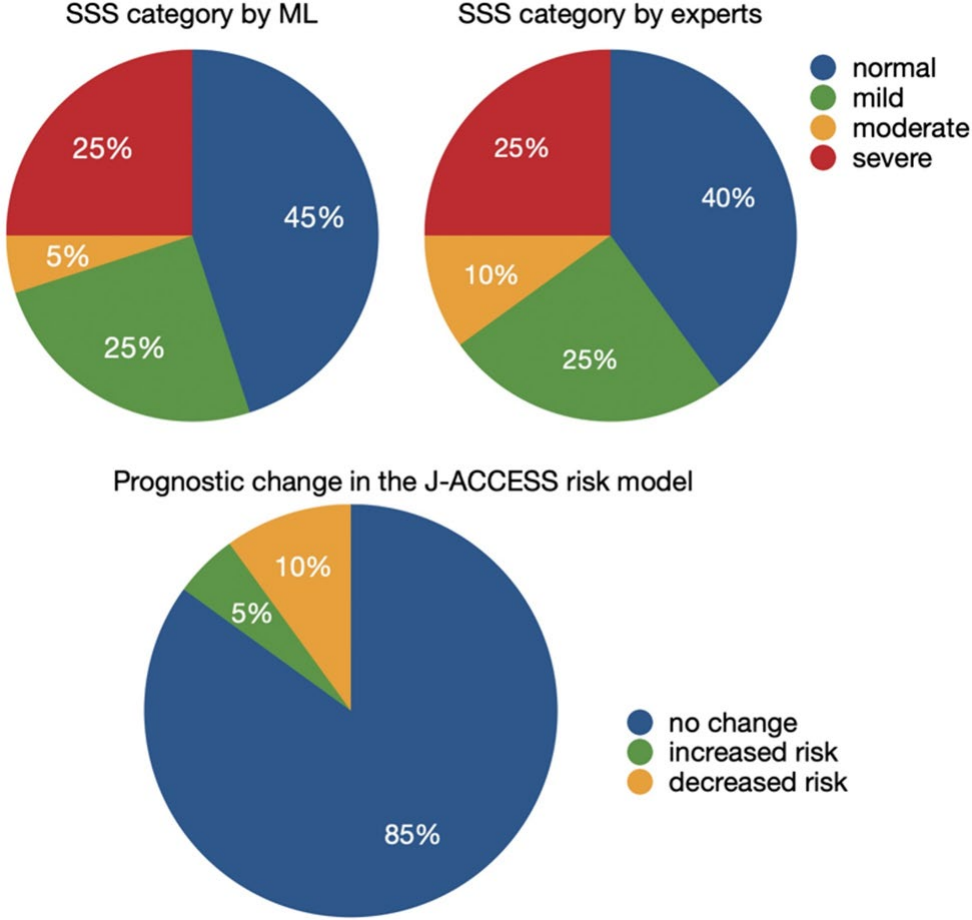
Comparison of SSS category between ML and experts. Change in prognostic risk levels with J-ACCESS risk model [23] is shown in the lower panel

In the J-ACCESS risk model, the SSS category is bifurcated into normal-mild vs. moderate-severe (0–3, normal; 4–7, mild; 8–11, moderate; ≥ 12, severe). Other four variables to calculate major cardia events, namely, age, summed stress score, left ventricular ejection fraction, and estimated glomerular filtration rate were the same in each patient [23]. Therefore, we compared the SSS category in test dataset (*n* = 20) between ML and J-ACCESS database, to estimate the prognostic significance of MPI values by ML (Fig. 5). Using this classification, 17 patients (85%) remained in the same SSS category, indicating no change in predicted prognosis. One patient (5%) showed an upward categorical change (from mild to moderate), while two patients (10%) showed a downward categorical change (from moderate to mild).

## Discussion

We created a new ML algorithm to estimate segmental scores of MPI using the J-ACCESS 3 and 4 databases that included scores assessed by nuclear cardiology experts. Overall, the ability of the ML algorithm to estimate defect scores was comparable to that of the experts. The accuracy of estimation for individual segments and total SSS, SRS, and SDS, was verified by comparison with that assessed by the experts.

The ability of ML to accurately estimate high scores indicating severe perfusion defects was lower than that for those indicating mild-moderate ischemia. We assumed that the J-ACCESS database had already excluded patients with myocardial infarction [10, 13]. This led to ML being unable to estimate higher scores because it could not extract features due to insufficient teaching data. Despite this, the accuracy of estimating lower scores indicating mild-moderate perfusion defects was preserved for clinical use.

In actual clinical practice, visual assessment seems to be relatively straightforward in patients with scores indicating severe perfusion defects, whereas areas of milder hypoperfusion are often difficult to evaluate. Since this algorithm can accurately estimate segments with mild to moderate hypoperfusion, it should be useful in clinical practice. Furthermore, estimations of SSS, SRS, and SDS were similar to those by the experts. Therefore, this would be useful for evaluating severity and identifying indications for treatments.

The complete segment-by segment concordance, underestimated, and overestimated rates between expert scores and those estimated by ML were 79.6%, 10.3%, and 10.0%, respectively. We speculated that these discrepancies arose because scores estimated in basal regions might be inaccurate, since determining the LV mitral valve plane by automated segmentation software could be difficult and variable [25–28]. However, the results showed that the estimated scores in the basal regions were more accurate than those in other regions. On the other hand, the agreement of segments 4, 5, 10, and 11 in the inferolateral regions was < 70%. Attenuation artifacts can be visually differentiated when all other information, such as wall motion, the condition of the edge in hypoperfused area is integrated. However, ML might have been unable to distinguish artifacts due to insufficient feature extraction from the training data that did not include functional data. Most of the segments showing higher concordance rates were located in basal regions as described above. Since these regions generally have a lower prevalence of positive findings compared to other regions, there might be a bias where regions with fewer positive scores tend to exhibit higher agreement rates.

Although there are concerns about the impact on the accuracy of ML estimation in terms of learning from images of various quality provided by multiple facilities in the J-ACCESS, the image acquisition and reconstruction conditions were separately surveyed during the multicenter study and were confirmed to follow the standard protocol recommendations. Therefore, it is presumed that the data do not include images that would significantly compromise the accuracy of the ML estimation.

In the J-ACCESS database, most image data were acquired by Anger-type gamma cameras. However, the prevalence of semiconductor detectors has been increasing recently. Since the segmental perfusion distribution acquired by semiconductor system is different from that by Anger-type system [29, 30], this difference might affect the ML score estimation. While the impact on AI may be less pronounced than on statistical data, it still needs to be verified by further investigation.

Several studies have determined the utility of AI for assessing MPI, and trends currently focus on risk assessment by combining clinical information with MPI findings [18–20, 22]. However, a scarcity of nuclear cardiology specialists in Japan has led to a demand for AI to help improve diagnostic accuracy. Under these circumstances, Nakajima et al. developed an algorithm using an artificial neural network (ANN) to assess of MPI [16, 17, 21]. The ANN was trained to classify potentially abnormal areas as true or false based on interpretations of ECG-gated and ungated stress/rest MPI by nuclear cardiology experts at several centers. They found that the diagnostic ability of ANN was comparable to that of expert interpretation. While they used ANN for the characterization of defects into ischemia and infarction with probability and extents, our study aimed to develop a new ML. Whereas the algorithms created by Nakajima et al. included the features of sex and cardiac function (wall motion and thickening) to train the ANN, the present algorithm was trained purely on perfusion distribution. As the J-ACCESS study already has a risk model for predicting cardiac events [6, 23], the ML estimation can also be applied to prognosis prediction. Therefore, the present ML algorithm should lead not only to more accurate evaluations of segmental scores, but also automated prognostic predictions.

### Limitations

The present study was a secondary analysis of the J-ACCESS database. Therefore, the new algorithm could not be sufficiently trained using data derived from females and scores for severe myocardial perfusion defect. In addition, the test and validation sets included relatively low numbers of patients. More data would improve training accuracy. Thus, further clinical studies are needed to verify the accuracy and usefulness of our algorithm.

## Conclusions

We developed a novel algorithm for estimating MPI scores using ML and data from the Japanese J-ACCESS 3 and 4 databases. Our algorithm should enable accurate MPI interpretation even in facilities without nuclear cardiology specialists, and support clinical practice in terms of therapeutic decision making and prognostic predictions.

## Data Availability

The datasets generated and/or analyzed in the current study are not publicly available due to approval of Ethics Committee.
